# The role of apoptosis and oxidative stress in the pathophysiology of *Acanthamoeba* spp. infection in the kidneys of hosts with different immunological status

**DOI:** 10.1186/s13071-023-06052-0

**Published:** 2023-12-01

**Authors:** Karolina Kot, Patrycja Kupnicka, Maciej Tarnowski, Patrycja Tomasiak, Danuta Kosik-Bogacka, Natalia Łanocha-Arendarczyk

**Affiliations:** 1 Department of Biology and Medical Parasitology, Pomeranian Medical University in Szczecin, Szczecin, Poland; 2 Department of Biochemistry, Pomeranian Medical University in Szczecin, Szczecin, Poland; 3 Department of Physiology in Health Sciences, Pomeranian Medical University in Szczecin, Szczecin, Poland; 4 Institute of Physical Culture Sciences, University of Szczecin, Szczecin, Poland; 5 Independent Laboratory of Pharmaceutical Botany, Department of Biology and Medical Parasitology, Pomeranian Medical University in Szczecin, Szczecin, Poland

**Keywords:** *Acanthamoeba* spp., Apoptosis, Immunological status, Kidneys, Oxidative stress

## Abstract

**Background:**

*Acanthamoeba* spp. are opportunistic pathogens that cause inflammation, mostly in the brain, lungs and cornea. Recent reports indicate kidney dysfunction in hosts with systemic acanthamoebiasis. The aim of the study was to analyze the gene expression and protein concentration of NADPH oxidase 2 and 4 (NOX2 and NOX4, respectively) and nuclear erythroid 2-related factor (Nrf2) in the kidneys of hosts with systemic acanthamoebiasis. We also aimed to determine the protein and gene expressions of Bcl2, Bax, caspases 3 and 9.

**Methods:**

Mice were divided into four groups based on their immunological status and *Acanthamoeba *sp. infection: A, immunocompetent *Acanthamoeba* sp.-infected mice; AS, immunosuppressed *Acanthamoeba* sp.- infected mice; C, immunocompetent uninfected mice; CS, immunosuppressed uninfected mice. NOX2, NOX4 and Nrf2 were analyzed by quantitative reverse transcription PCR (qRT-PCR) and ELISA methods, while pro-apoptotic and anti-apoptotic proteins (Bax and Bcl-2, respectively), Cas9, Cas3 were analyzed by qRT-PCR and western blot methods.

**Results:**

Increased gene expression and/or protein concentration of NOX2 and NOX4 were found in both immunocompetent and immunosuppressed mice infected with *Acanthamoeba* sp. (groups A and AS, respectively). Gene expression and/or protein concentration of Nrf2 were higher in group A than in control animals. Compared to control mice, in the AS group the expression of the Nrf2 gene was upregulated while the concentration of Nrf2 protein was decreased. Additionally in A group, higher gene and protein expression of Bcl-2, and lower gene as well as protein expression of Bax, caspases 3 and 9 were noted. In contrast, the AS group showed lower gene and protein expression of Bcl-2, and higher gene as well as protein expression of Bax, caspases 3 and 9.

**Conclusions:**

This study is the first to address the mechanisms occurring in the kidneys of hosts infected with *Acanthamoeba* sp. The contact of *Acanthamoeba* sp. with the host cell surface and/or the oxidative burst caused by elevated levels of NOXs lead to an antioxidant response enhanced by the Nrf2 pathway. *Acanthamoeba* sp. have various strategies concerning apoptosis. In immunocompetent hosts, amoebae inhibit the apoptosis of kidney cells, and in immunosuppressed hosts, they lead to increased apoptosis by the intrinsic pathway and thus to a more severe course of the disease.

**Graphical Abstract:**

**Figure:**
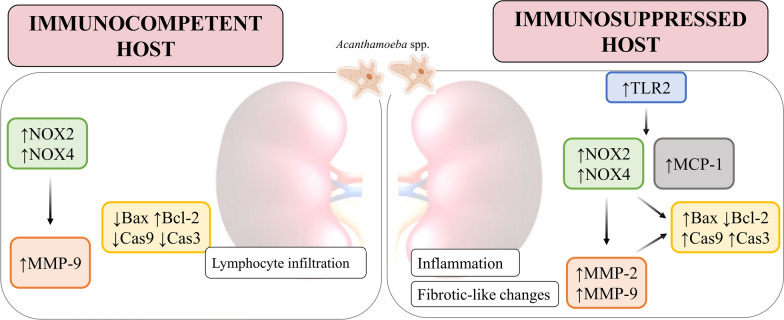

## Background

*Acanthamoeba* spp. are protozoa with pathogenic properties that are ubiquitously distributed in the environment. They occur in water, soil and air in the form of metabolically active trophozoites and resistant cysts, which enable the amoebae not only to survive without nutrients but also to resist disinfection and treatment measures [1]. The only exception is *Acanthamoeba pyriformis* which additionally includes facultative sporocarp fruiting in its life-cycle [2]. These amoebae are the etiological factor of granulomatous amebic encephalitis, amoebic keratitis (AK) and systemic acanthamoebiasis, following invasion of various tissues and organs [3]. Disseminated acanthamoebiasis is characterized by multi-organ and multi-symptom lesions with a rapid course leading to the death of the host. The average time of infection for a patient with systemic acanthamoebiasis, based on current literature, is 18 months (range: 3 months to 6 years) [4]. *Acanthamoeba* spp. infection in the kidneys has been described only in one patient in whom urinalysis showed no abnormalities [5]. However, there is no data on the true number of patients with kidneys affected by amoebae. Many clinical cases described in the scientific literature do not report whether the kidneys were studied for the presence of amoebae during post-mortem examinations [6–9]. Animal studies, on the other hand, have clearly revealed that amoebae are transported in the blood system and penetrate the kidneys where they can cause dysfunction [10]. Understanding the pathomechanisms of infection through biochemical and molecular studies of the *Acanthamoeba* spp.-host system is essential for the development of new diagnostic procedures and the identification of new therapeutic targets that will limit the degree of cell and organ damage.

One of the mechanisms responsible for kidney dysfunction is oxidative stress, which is defined as an imbalance between the generation of reactive oxygen species (ROS) and the antioxidant capacity of the organism [11, 12]. Excessive ROS impair cellular homeostasis and function through cell death, leading to inflammation, tissue damage and fibrosis [13]. One mechanism by which ROS are produced is by the NADPH oxidase family of enzymes, among which NADPH oxidase 2 (NOX2) and NADPH oxidase 4 (NOX4) are highly expressed in the kidneys [14]. NOX4 plays a dual role in the kidneys. Under physiological conditions, this enzyme mediates a steady redox signal that favors cellular quiescence and differentiation. Complete loss of NOX4 function leads to inefficient oxidation of protein targets, resulting in the cell becoming more susceptible to challenges by stressors and, ultimately, leading to cell damage. In contrast, overexpression of NOX4 leads to increases in H_2_O_2_ levels, possibly resulting in oxidative damage to proteins and ultimately cell death [15, 16]. These findings have led to the assumption that NOX4 upregulation promotes cellular damage and kidney disease progression [17]. To protect cells from harmful oxidative stress, the nuclear erythroid 2-related factor (Nrf2) plays an integrative role in inducing the expression of genes encoding enzymes involved in antioxidant production and in the reduction of pro-oxidants. In cells free of oxidative stress, the newly synthesized Nrf2 protein is directly captured by the stress-sensor molecule Keap1 and degraded through the ubiquitin–proteasome pathway. Under oxidative stress conditions, Nrf2 avoids Keap1-mediated degradation because Keap1 is denatured by ROS [13, 18]. If the antioxidative system is insufficient, apoptosis can occur. Free radicals, lack of cytokines and viral infections activate the internal pathway of apoptosis, which is called the mitochondrial pathway. These stimuli cause modifications in the inner mitochondrial membrane, resulting in the release of apoptotic proteins. Pro-apoptotic proteins include Bax, Bcl-10, Bik, Bak, Blk, Bad, Bim, Puma and Noxa, while anti-apoptotic proteins include Bcl-2, Bag, Bcl-XS, Bcl-XL, Bcl-x and Bcl-1 [19]. Bax and Bak proteins, as a result of oligomerization, form channels in the mitochondrial membrane and are thus responsible for the release of mitochondrial contents into the cytoplasm. If the apoptosis-inducing factor is missing, Bax and Bak are inhibited by proteins with anti-apoptotic properties, such as Bcl-2, Bcl-XL. The ratios of Bax and Bcl-2 are correlated with the release of cytochrome* c* and subsequent activation of the caspase cascade. The first step in this activation process is activation of the initiator enzyme caspase 9 (Cas9), which then activates the effector enzyme caspase 3 (Cas3) [20]. Cas3 cleavage ultimately leads to the breakdown of DNA and degradation of both cytoskeletal and nuclear proteins [19]. Apoptotic bodies are also formed and ligands for phagocytic cell receptors are expressed [20]. In a healthy and mature kidney, apoptosis is observed at a relatively low degree, but it can be significantly intensified in the presence of kidney damage, leading to the development of many diseases of this organ [21].

The aim of this study was to analyze the effect of *Acanthamoeba* spp. on the formation of oxidative stress (assessment of gene expression and protein concentration of NOX2, NOX4 and Nrf2) and on the apoptosis process (assessment of protein and gene expression of Bcl2, Bax, Cas3 and Cas9) in kidneys of laboratory animals. Since *Acanthamoeba* spp. are classified as opportunistic parasites, both immunocompetent and immunosuppressed hosts were used in the study.

## Methods

### *Acanthamoeba* strain

The *Acanthamoeba* sp. used in the present study is a clinical strain (AM22 strain) isolated in 2007 from a bronchoaspirate of a patient with hematopoietic malignancy. Genetic analyses allowed the strain to be classified as the T16 genotype (GenBank reference number: GQ342607) [22]. Amoebae were maintained in vitro on non-nutrient agar plates at 37 °C.

### Animal model

**Fig. 1:**
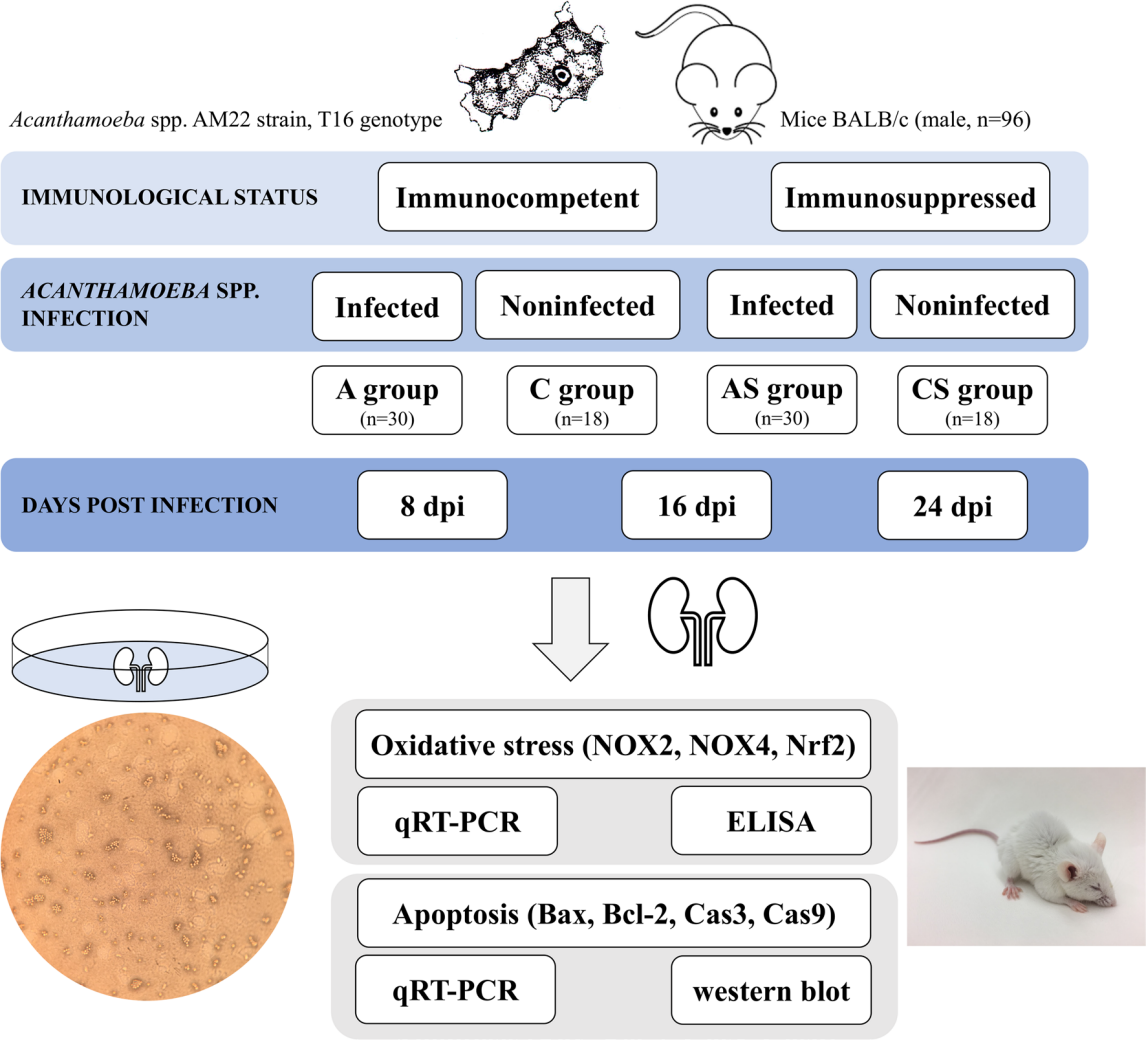
Scheme of the experiment. *Acanthamoeba* sp. (AM22 strain) used in the study belongs to genotype T16. Mice (*n* = 96) were divided into two groups for the experiment: immunocompetent (groups A and C) and immunosuppressed (groups AS and CS). Immunosupression of mice from AS and CS groups was achieved by administering methylprednisolone intraperitoneally for 4 days prior to amoebae inoculation. Mice from both the immunosuppressed and immunocompetent groups were each then randomly divided into two groups: infected (groups AS and A, respectively) and uninfected with amoebae (groups CS and C, respectively). Mice from groups A and AS (infected groups) were infected by intranasal administration of 3 µl of a suspension containing amoebae trophozoites, while mice from groups C and CS (uninfected groups) received 3 µl of saline. Animals were sacrificed on days 8, 16 and 24 post *Acanthamoeba* sp. infection and the kidneys were removed. A fragment of each kidney was placed on non-nutrient agar to re-isolate amoebae; the remaining kidney tissue was kept at − 80 °C. NOX2, NOX4 and Nrf2 were analyzed by qRT-PCR and ELISA methods. Pro-apoptotic and anti-apoptotic proteins (Bax and Bcl-2, respectively), Cas9 and Cas3 were analyzed by qRT-PCR and western blot. Cas3, Caspase 3; Cas9, caspase 9; dpi, days post *Acanthamoeba* sp. infection; ELISA, enzyme-linked immunosorbent assay; NOX2, NADPH oxidase 2; NOX4, NADPH oxidase 4; Nrf2, nuclear erythroid 2-related factor; qRT-PCR, quantitative reverse transcription PCR

The animal model has been described previously [10, 23]. Briefly, 96 male BALB/c mice, 6–10 weeks of age, were used in the study. The mice were divided into groups as shown in Fig. 1. Immunosuppressed mice were divided into two groups: *Acanthamoeba* sp.-infected mice (AS) and *Acanthamoeba* sp.-uninfected control mice (CS). They were then administered 220 µl of methylprednisolone sodium succinate (MPS; Solu-Medrol; Pfizer, New York, NY, USA) dissolved in 0.1 ml of 0.1% saline to suppress their immune response. The drug was administered intraperitoneally (i.p.) to the mice at 96, 72, 48, and 24 h before infection with *Acanthamoeba* sp. The model of immunosuppressive drug administration was designed based on the scientific literature [24]. Infected mice, both immunocompetent *Acanthamoeba* sp.-infected mice (group A) and immunosuppressed *Acanthamoeba* sp.-infected mice (group AS) were infected by intranasal administration of 3 µl of a suspension containing 10,000–20,000 amoebae trophozoites; uninfected mice, namely immunocompetent uninfected mice (C group) and immunosuppressed uninfected mice (CS group) received 3 µl of saline. Animals were sacrificed on days 8, 16 and 24 post *Acanthamoeba *sp. infection (dpi) by administration of sodium pentobarbital i.p. (Euthasol vet, Raamsdonksveer, The Netherlands; 2 ml/kg body weight) and then necropsied. Kidneys were removed from the mice using sterile equipment, fixed in liquid nitrogen and stored at −80 °C until further analysis. The invasion of *Acanthamoeba *sp. into the kidneys was checked by re-isolating the amoebae from a fragment of the kidneys. Animals from which amoebae were not re-isolated from the kidneys were excluded from further studies on the mechanisms occurring in the kidneys of hosts with systemic acanthamoebiasis.

### Analysis of Bax, Bcl-2, Cas3, Cas9, NOX2, NOX4 and Nrf2 gene expressions by qRT-PCR

Quantification of the expression of messenger RNA (mRNA) of the Bcl-2, Bax, Cas3, Cas9, NOX2, NOX4 and Nrf2 genes was performed by two-step quantitative real-time PCR (qRT-PCR). The relative expression of the studied genes was determined in relation to the average expressions of the glyceraldehyde 3-phosphate dehydrogenase (*Gapdh*) and beta-2 microglobulin reference (*B2M*) genes with constitutive expression (housekeeping genes). Total RNA was isolated from frozen kidneys using the RNeasy Mini Kit (Qiagen, Hilden, Germany) according to the manufacturer's instructions. The concentration and purity of the isolated RNA were determined using a Nanodrop ND-1000 spectrophotometer (Thermo Fisher Scientific, Waltham, MA, USA), following which 1 μg of tissue-isolated RNA was prepared for analysis using a First Strand cDNA Synthesis Kit and oligo-dT primers (Thermo Fisher Scientific) (Table 1). Real-time quantification of mRNA levels was performed using the 7500 Fast Real-Time PCR System (Applied Biosystems, Thermo Fisher Scientific) with Power SYBR Green PCR Master Mix (Applied Biosystems, Thermo Fisher Scientific).

**Table 1:** Primers used in quantitative real-time PCR analyses

Gene	Forward	Reverse
Gapdh	GGA GAA ACC TGC CAA GTA TGA TG	GAC AAC CTG GTC CTC AGT GTA GC
B2M	CATACG CCT GCA GAG TTA AGC A	GAT CAC ATG TCT CGA TCC CAG TAG
Bcl-2	GTC CCG CCT CTT CAC CTT TCA G	GAT TCT GGT GTT TCC CCG TTG G
Bax	GCG TGG TTG CCC TCT TCT ACT TTG	AGT CCA GTG TCC AGC CCA TGA TG
Cas3	ATG GAG AAC AAC AAA ACC TCA GT	TTG CTC CCA TGT ATG GTC TTT AC
Cas9	GGC TGT TAA ACC CCT AGA CCA	TGA CGG GTC CAG CTT CAC TA
NOX2	AGT GCG TGT TGC TCG ACA A	GCG GTG TGC AGT GCT ATC AT
NOX4	AGT CAA ACA GAT GGG A	TGT CCC ATA TGA GTT GTT
Nrf2	TAG ATG ACC ATG AGT CGT TGC	GCC AAA CTT GCT CCA TGT CC

*B2M* Beta-2 microglobulin,*Cas3* Caspase 3,* Cas9* caspase 9,* Gapdh* glyceraldehyde 3-phosphate dehydrogenase,* NOX2* NADPH oxidase 2,* NOX4* NADPH oxidase 4,* Nrf2* nuclear erythroid 2-related factor

### Analysis of Bax, Bcl-2, Cas3, Cas9 protein expressions

Protein electrophoresis was performed using a sodium dodecyl sulfate-polyacrylamide gel electrophoresis (PAGE) system which consisted of both a lower (14% separating gel) and upper (3% thickening gel) layer. Samples were lysed in RIPA buffer (Cell Signaling Technology, Danvers, MA, USA [cat. no.: 9806]) with protease and phosphatase inhibitors (PhosSTOP™; Roche, Basel, Switzerland [cat. no.: 4906845001]; cOmplete™; Roche, Switzerland [cat. no.: 11836153001]). Each sample contained 30 μg of protein. Sample buffer (4× Laemmli Sample Buffer; BioRad Laboratories, Hercules, USA [cat. no.: 1610747]) mixed with 2-mercaptoethanol (Sigma-Aldrich, St. Louis, MO, USA [cat. no.: M3148]) was added to each sample in accordance with the manufacturer's recommendations, and the samples then heated for 5 min at 95 °C.

The electrophoresis of proteins and standard markers (Precision Plus Protein™ All Blue Prestained Protein Standards; BioRad Laboratories) was carried out in a buffer-filled SDS-PAGE system (1 l distilled water, 14.4 g glycine, 3.03 g Trizma Base, 5.0 g SDS) for 15 min at 100 V and 90 min at 130 V.

Then,  the "transfer sandwich" was prepared,  closed and placed in the transfer cassette located in the tub of the system. The wet transfer method was used, i.e. the fractionated proteins were transferred to a 0.2-µm PVDF membrane (Thermo Fisher Scientific) at 75 V for 60 min, following which the membrane was blocked with 5% bovine serum albumin in a blocking buffer for 1 h at room temperature. The expression of apoptotic proteins was determined by immunodetection with specific antibodies. Primary monoclonal antibodies against Cas3 (Abcam, Cambridge, UK [cat. no.: ab13585-100]) and Cas9 (Abcam [cat. no.: ab184786-100]) and polyclonal antibodies against Bax (Abcam [cat. no.: ab196494-100]) and Bcl-2 (Abcam [cat. no.: ab196495-100]), at a dilution of 1:500, were used. After being placed in primary antibodies, the membrane was incubated overnight at refrigerator temperature. Then, the membrane was rinsed and it was  incubated with a secondary anti-mouse antibody (Abcam [cat. no.: ab6789-1]) or an anti-rabbit antibody (Abcam [cat. no.: ab205718]) at a dilution of 1:10000 for 1 h at room temperature. The ECL Advance Western Blotting Detection Kit (GE Healthcare, Chicago, IL, USA) was used to visualize protein expression, following which the bands were developed using the ChemiDock XRS + Molecular Imager (Bio-Rad Laboratories). Densitometric analysis was performed using Image Lab Software version 6.1.0 (Bio-Rad Laboratories). Alpha tubulin (Abcam [cat. no.: 7291]) was used as a reference protein.

### Measurement of NOX2, NOX4, and Nrf2 concentrations

NOX2 concentration was determined using the Mouse Nicotinamide Adenine Dinucleotide Phosphate Oxidase 2 (NOX2) ELISA Kit (EIAab, Wuhan, China), and NOX4 and Nrf2 concentrations were determined using the Mouse NADPH Oxidase 4 ELISA Kit and Mouse Nuclear Factor Erythroid 2-related Factor 2 (NRF2) ELISA Kit, respectively (both BT LAB, Shanghai, China). The assays were performed according to the respective manufacturer’s recommendations, and the results were measured using the EZ Read 2000 microplate reader (Biochrom Ltd., Cambridge, UK).

### Statistical analysis

Statistical analysis was performed using StatSoft Statistica version 8.0 (TIBCO Software Inc., Palo Alto, CA, USA) and GraphPad version 4.0 (GraphPad Software Inc., San Diego, CA, USA). For each of the studied parameters, the arithmetic mean and the standard deviation from the arithmetic mean were calculated. As the data did not follow a normal distribution (according to the Shapiro–Wilk test), differences between the studied parameters were calculated by using the non-parametric Mann-Whitney U-test and Kruskal-Wallis H-test. The* p* < 0.05 level was taken as a significant statistical difference.

## Results

### Effects of *Acanthamoeba* sp. on NOX2, NOX4 and Nrf2 gene expression in the mouse kidneys

**Fig. 2:**
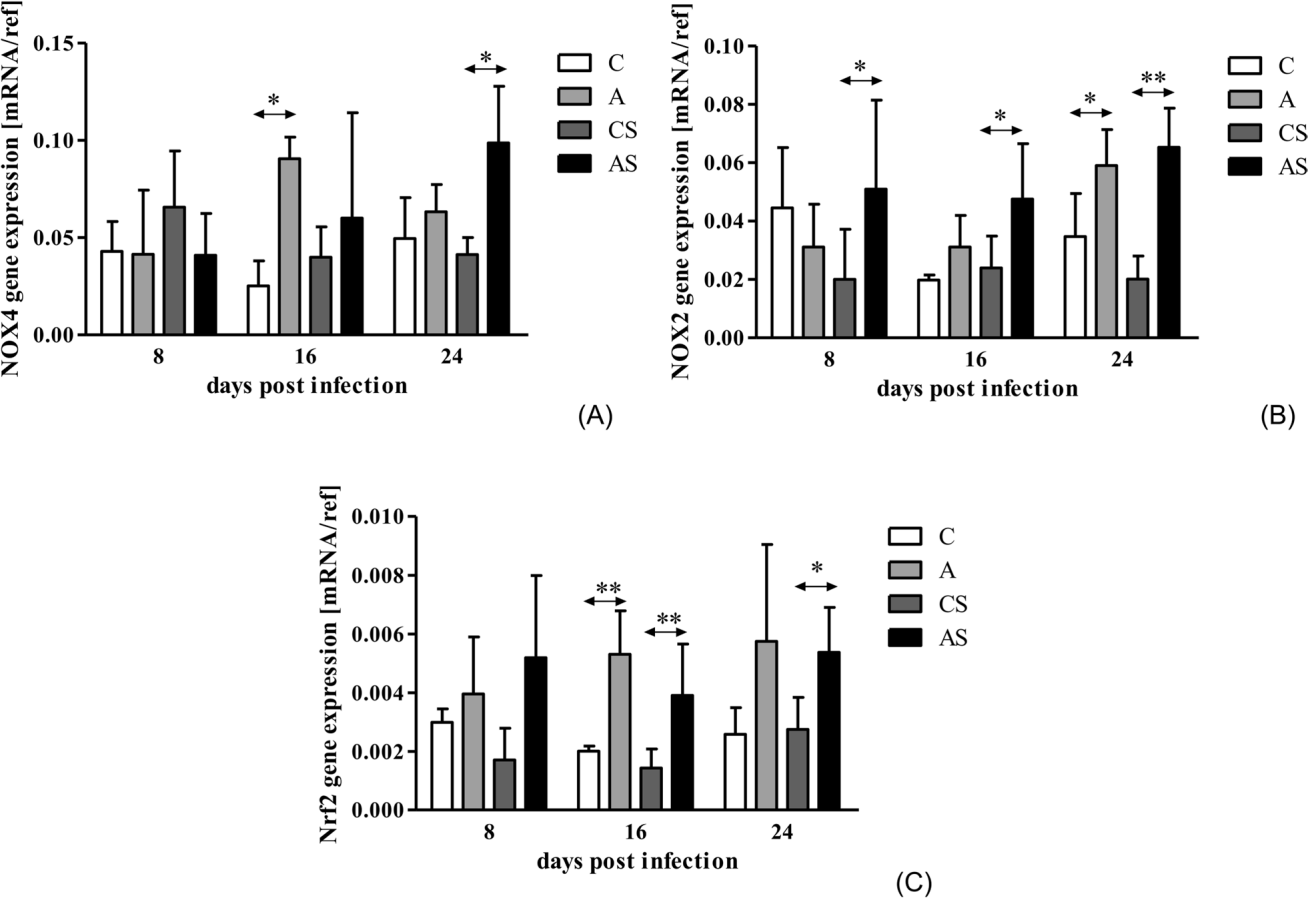
The gene expression of NOX4 (**A**), NOX2 (**B**), and Nrf2 (**C**) in the hosts’ kidneys. Gene expressions were determined using qRT-PCR. *Gapdh* and* B2M* were used as reference genes. Data represent the arithmetic mean ± standard deviation for 6 independent experiments. Asterisks indicate statistically significant differences at *p < 0.05 and **p < 0.01. A, Immunocompetent group infected with *Acanthamoeba* sp.; AS, immunosuppressed group infected with *Acanthamoeba* sp.;* B2M*, beta-2 microglobulin gene; C, immunocompetent noninfected group; CS, immunosuppressed noninfected group;* Gapdh*, glyceraldehyde 3-phosphate dehydrogenase gene; mRNA, messenger RNA; NOX2, NADPH oxidase 2; NOX4, NADPH oxidase 4; Nrf2, nuclear erythroid 2-related factor; qRT-PCR, quantitative real-time PCR; ref; reference genes

In A group, we observed  an increase in NOX4 gene expression at 16 dpi compared to the control group (Mann-Whitney U-test; *U* = 0.00, *p* = 0.02; Fig. 2A). Moreover, we noted  a statistically significant difference in NOX4 gene expression between days post *Acanthamoeba* sp. infection in group A (Kruskal-Wallis H-test; *H* = 7.71, *p* = 0.02). In AS group, there was a statistical significant increase in NOX4 gene expression at 24 dpi compared to the CS group (Mann-Whitney U-test; *U* = 0.00, *p* = 0.004). Additionally, there was a statistically significant difference in NOX4 gene expression between days post *Acanthamoeba* sp. infection in the AS group (Kruskal-Wallis H-test; *H* = 6.23, *p* = 0.04).

NOX2 gene expression was increased in A group at 24 dpi compared to the control group (Mann-Whitney U-test; *U* = 1.00, *p* = 0.02). In AS group, upregulation of NOX2 gene expression was observed at the beginning of infection compared to the CS group (Mann-Whitney U-test; *U* = 5.00; *p* = 0.04; Fig. 2B). Moreover, NOX2 gene expressions were increased also at 16 dpi (Mann-Whitney U-test; *U* = 4.00, *p* = 0.02) and 24 dpi in the AS group (Mann-Whitney U-test, *U* = 0.00, *p* = 0.004; Fig. 2B). Comparing days post infection, there was a statistically significant difference between 8 versus 16 versus 24 dpi only in the A group (Kruskal-Wallis H-test;* H* = 7.79; *p* = 0.02).

Nrf2 gene expression was upregulated in group A at 16 dpi compared to the control group (Mann-Whitney U-test; *U* = 0.00, *p* = 0.004). In the AS group, Nrf2 gene expression was upregulated at both 16 and 24 dpi compared to group CS (Mann-Whitney U-test; *U* = 2.00; *p* = 0.008 and *U* = 2.00; *p* = 0.01, respectively; Fig. 2C).

### Effects of *Acanthamoeba* sp. on NOX2, NOX4 and Nrf2 protein concentration in the mouse kidneys

**Fig. 3:**
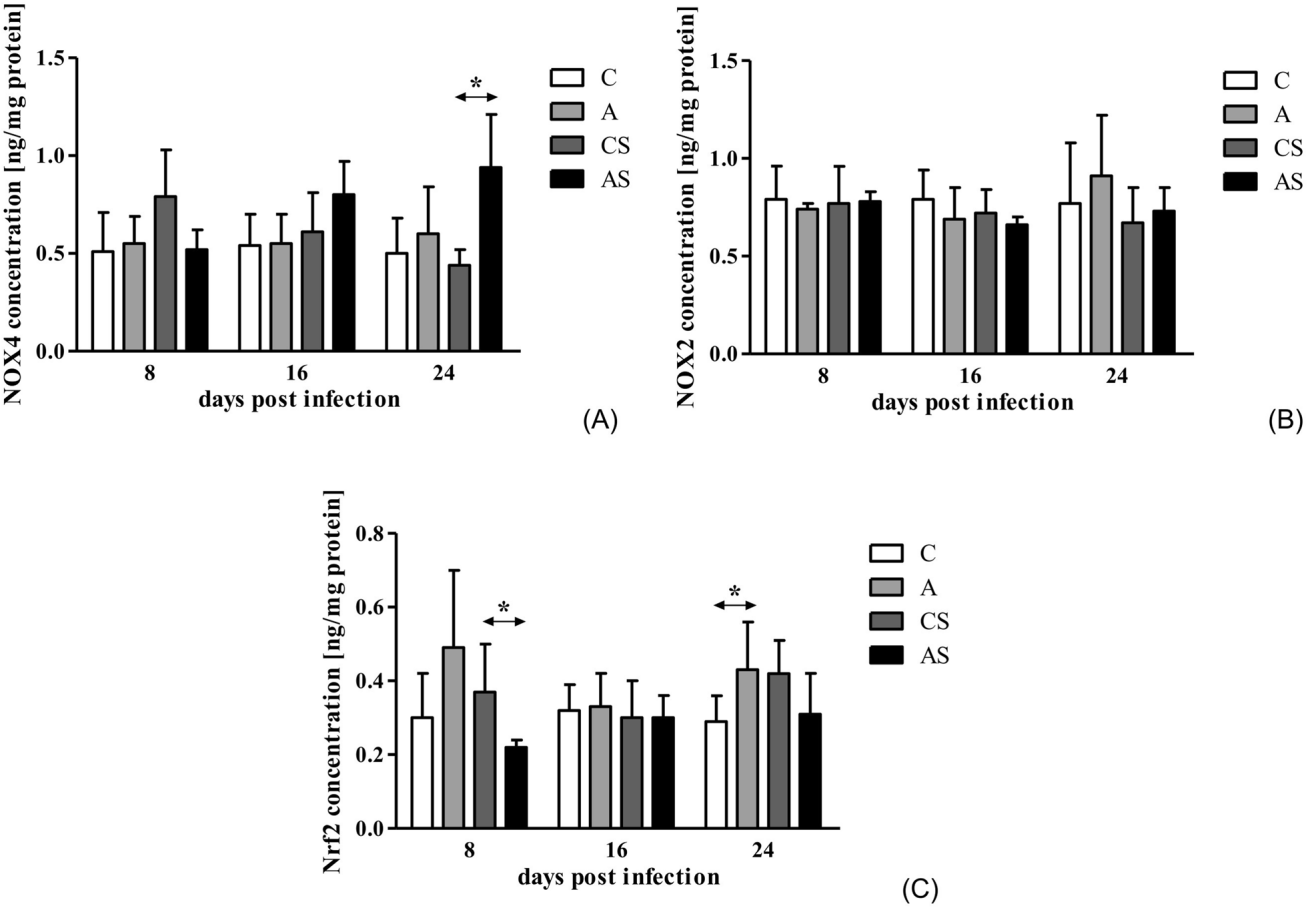
Concentration of NOX4 (**A**), NOX2 (**B**) and Nrf2 (**C**) protein in the hosts’ kidneys. Protein concentrations were analyzed by ELISA. Data represent the arithmetic mean ± standard deviation for 6 independent experiments. Asterisks indicate statistically significant differences at *p < 0.05. A, Immunocompetent group infected with *Acanthamoeba* sp.; AS, immunosuppressed group infected with *Acanthamoeba* sp.; C, immunocompetent noninfected group; CS, immunosuppressed noninfected group; NOX2, NADPH oxidase 2; NOX4, NADPH oxidase 4; Nrf2, nuclear erythroid 2-related factor

The concentration of NOX4 in group A was similar on each day of the infection. In comparison, in the AS group, NOX4 protein concentration increased at 24 dpi compared to the control group (Mann-Whitney U-test; *U* = 0.00; *p* = 0.03; Fig. 3A). There was a statistically significant difference between days post *Acanthamoeba* sp. infection in the AS group (Kruskal-Wallis H-test;* H* = 7.84, *p* = 0.02).

NOX2 concentration in A and AS groups was similar on each day of the infection. Immunoenzymatic analysis showed no statistically significant changes in NOX2 protein concentration in the kidneys of mice (Fig. 3B).

We noted a higher concentration of Nrf2 in the A group at 8 and 24 dpi compared to the C group, but a statistically significant difference was observed only at 24 dpi (Mann-Whitney U-test; *U* = 2.00, *p* = 0.04; Fig. 3C). In AS group at 8 dpi, Nrf2 concentration was lower than in the CS group (Mann-Whitney U-test; *U* = 1.00, *p* = 0.05). A decreased Nrf2 concentration was also noted in the AS group at 24 dpi compared to control mice, but the difference was not statistically significant.

### Effects of *Acanthamoeba* sp. on Bax, Bcl-2, Cas3 and Cas9 gene expression in the mouse kidneys

**Fig. 4:**
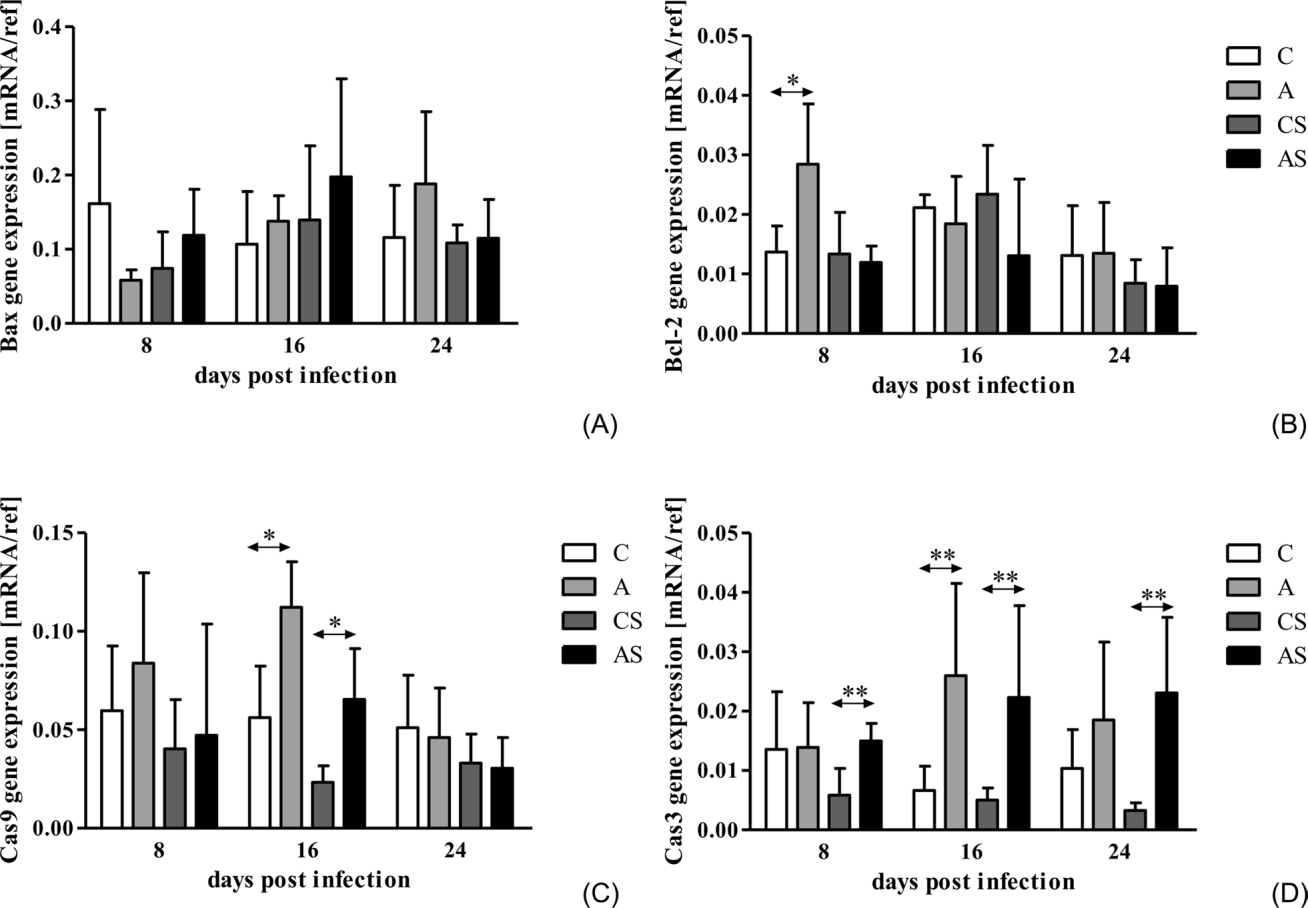
Gene expression of Bax (**A**), Bcl-2 (**B**), Cas9 (**C**) and Cas3 (**D**) in the hosts’ kidneys. Gene expressions were determined using qRT-PCR. *Gapdh* and *B2M* were used as reference genes. Data represent the arithmetic mean ± standard deviation for 6 independent experiments. Asterisks indicate statistically significant differences at *p < 0.05 and **p < 0.01. A, Immunocompetent group infected with *Acanthamoeba* sp.; AS, immunosuppressed group infected with *Acanthamoeba* sp.;* B2M*, beta-2 microglobulin gene; C, immunocompetent noninfected group; Cas3, caspase 3; Cas9, caspase 9; CS, immunosuppressed noninfected group;* Gapdh*, glyceraldehyde 3-phosphate dehydrogenase gene; mRNA, messenger RNA; qRT-PCR, quantitative real-time PCR; ref; reference genes

There was no statistically significant difference in the expression of the proapoptotic Bax gene in immunocompetent and immunosuppressed infected mice compared to the respective controls (Fig. 4A). However, statistically significant differences were noted in Bax gene expression between different days post infection in immunocompetent mice (Kruskal-Wallis H-test;* H* = 11.13, *p* = 0.004). We found no statistically significant difference in Bax gene expression in the days following amoebae infection in immunosuppressed animals.

Gene expression of the antiapoptotic Bcl-2 gene was increased in group A at 8 dpi compared to group C (Mann-Whitney U-test; *U* = 3.00; *p* = 0.03; Fig. 4B). In immunosuppressed mice, Bcl-2 gene expression was similar at all time points (Fig. 4B). Comparison of days post infection revealed that there were no significant differences in Bcl-2 gene expression at 8 versus 16 versus 24 dpi in both immunocompetent and immunosuppressed infected mice.

Gene expression of Cas9 (apoptosis initiator) at 16 dpi was higher in group A than in group C (Mann-Whitney U-test; *U* = 1.00, *p* = 0.02; Fig. 4C). Cas9 gene expression was also increased at 16 dpi in the AS group compared to the CS group (Mann-Whitney U-test; *U* = 1.00, *p* = 0.02; Fig. 4C). We noted a statistically significant difference in Cas9 gene expression between different days post infection in immunocompetent mice (Kruskal-Wallis H-test;* H* = 7.00, *p* = 0.03). In comparison, the differences in Cas9 gene expression between 8 versus 16 versus 24 dpi in the immunosuppressed infected mice were insignificant.

Gene expression of Cas3 (effector, executioner) was higher at 16 dpi in group A than in group C (Mann-Whitney U-test; *U* = 0.00, *p* = 0.008; Fig. 4D). In immunosuppressed mice, Cas3 gene expression was increased at 8 dpi (Mann-Whitney U-test; *U* = 1.00, *p* = 0.004), 16 dpi (*U* = 0.00, *p* = 0.003) and 24 dpi compared to group CS (*U* = 0.00; *p* = 0.006; Fig. 4D). There was a visible  difference in Cas3 gene expression at 8 versus 16 versus 24 dpi in the immunocompetent mice, but these differences were not statistically significant. In immunosuppressed mice, the differences in Cas3 gene expression at 8 versus 16 versus 24 dpi were also not statistically significant.

### Effects of *Acanthamoeba* sp. on Bax, Bcl-2, Cas3 and Cas9 protein expression in the mouse kidneys

**Fig. 5:**
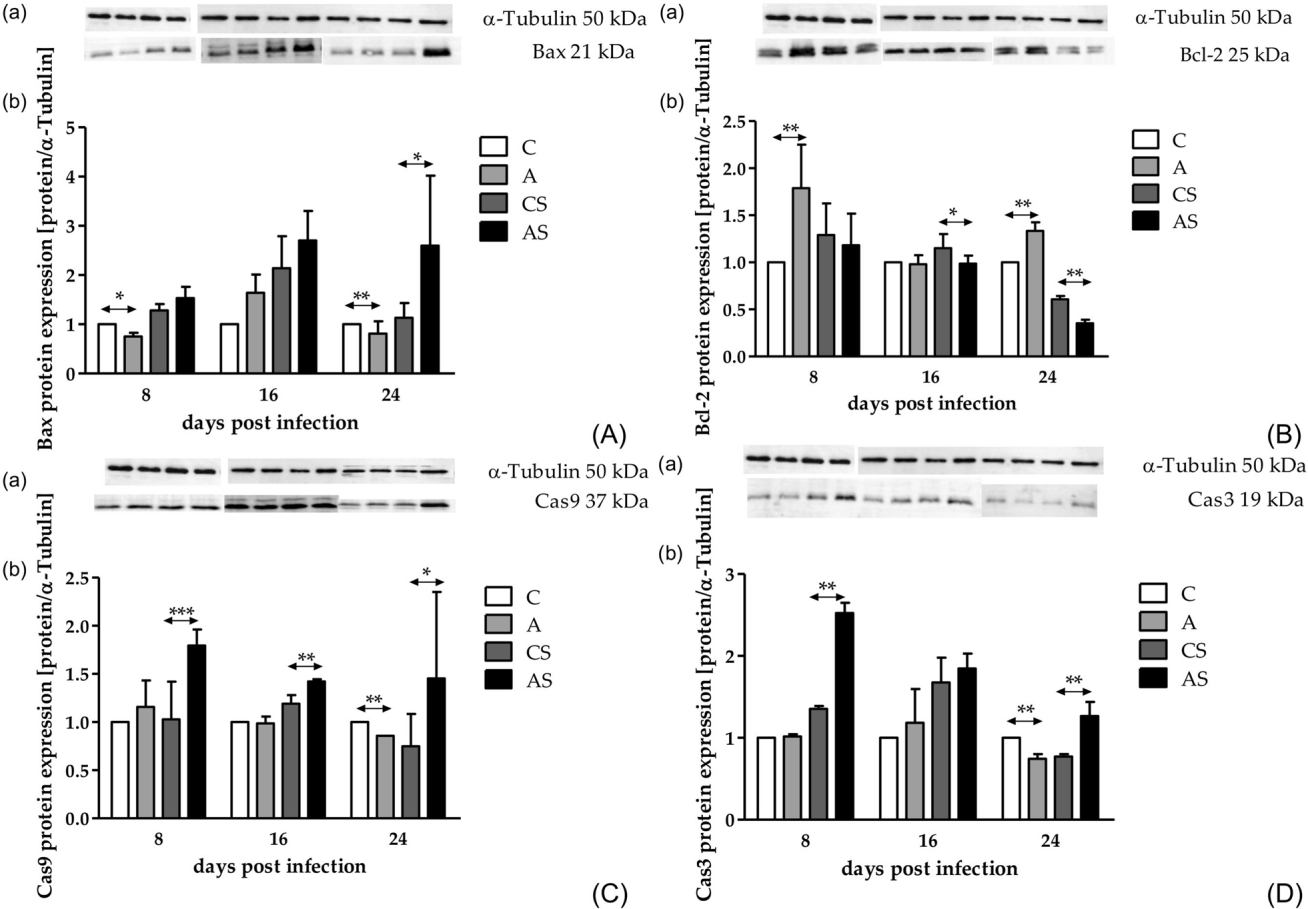
Protein expression of Bax (**A**), Bcl-2 (**B**), Cas9 (**C**) and Cas3 (**D**) in the hosts’ kidneys. Proteins were determined by western blot. Representative western blot (**a**) and densitometric analyses of protein (**b**) are shown. Data represent the arithmetic mean ± standard deviation for 6 independent experiments. Asterisks indicate statistically significant differences at *p < 0.05, **p < 0.01 and ***p < 0.001. A, Immunocompetent group infected with *Acanthamoeba* sp.; AS, immunosuppressed group infected with *Acanthamoeba* sp.; C, immunocompetent noninfected group; Cas3, caspase 3; Cas9, caspase 9; CS, immunosuppressed noninfected group

Bax protein expression was significantly lower in immunocompetent mice at 8 and 24 dpi compared to control mice (Mann-Whitney U-test; *U* = 0.00; *p* = 0.002 and *U* = 0.00; *p* < 0.001, respectively). In immunosuppressed mice infected with *Acanthamoeba* sp. (group AS), Bax protein expression was higher at all time points compared to uninfected mice, but only at 24 dpi was the difference statistically significant (Mann-Whitney U-test; *U* = 12.00, *p* = 0.002; Fig. 5A). Taking into account days post infection, there was a statistically significant difference in Bax protein expression in immunocompetent infected mice at 8 versus 16 versus 24 dpi (Kruskal-Wallis H-test;* H* = 12.02; *p* = 0.0024); in contrast, in immunosuppressed mice, differences in Bax protein expression according to days post infection were not statistically significant.

Protein expression of the antiapoptotic Bcl-2 was statistically significant at 8 versus 16 versus 24 dpi in both immunocompetent and immunosuppressed infected mice (Kruskal-Wallis H-test;* H* = 14.78, *p* < 0.001 and* H* = 12.63, *p* = 0.002, respectively). Higher Bcl-2 protein expression was observed at 8 and 24 dpi in group A than in group C (Mann-Whitney U-test; *U* = 0.00, *p* = 0.002 and *U* = 0.00, *p* = 0.002; Fig. 5B). In comparison, in the AS group, we found a lower level of Bcl-2 protein expression at 16 and 24 dpi (Mann-Whitney U-test; *U* = 8.00; *p* = 0.01 and *U* = 0.00; *p* = 0.002, respectively) (Fig. 5B).

Cas9 protein expression was not statistically significant between 8, 16 and 24 dpi in both immunocompetent and immunosuppressed infected mice. Cas9 protein expression at 24 dpi was lower in group A compared to group C (Mann-Whitney U-test; *U* = 24.00, *p* = 0.004; Fig. 5C); in comparison, in AS group, Cas9 protein expression was higher at 8, 16 and 24 dpi (Mann-Whitney U-test; *U* = 0.00, *p* < 0.001; *U* = 0.00, *p* = 0.002; and *U* = 36.00, *p* = 0.04, respectively) (Fig. 5C).

Expression of the cleaved (activated) Cas3 protein was lower at 24 dpi in group A compared to group C (Mann-Whitney U-test; *U* = 0.00, *p* = 0.002; Fig. 5D). In immunosuppressed mice, Cas3 protein expression was increased at 8 dpi 24 dpi compared to the CS group (Mann-Whitney U-test; *U* = 0.00; *p* = 0.002 and *U *= 0.00; *p* = 0.002, respectively; Fig. 5D). Taking into account days post infection, in immunocompetent mice, protein expression of Cas3 differed at 8 versus 16 versus 24 dpi (Kruskal-Wallis H-test;* H* = 6.56; *p* = 0.04). In immunosuppressed mice, Cas3 protein expression was downregulated with increasing duration of the infection (Kruskal-Wallis H-test;* H* = 16.93, *p* < 0.001).

## Discussion

*Acanthamoeba* spp. are able to circulate in the blood of the host and thus migrate to and invade any organ, including the kidneys. In a previous study, among immunocompetent and immunosuppressed mice infected intranasally with *Acanthamoeba* sp., amoebae were re-isolated from the kidneys of 60% and 57% of animals, respectively [25]. Pathomorphological studies of the kidneys of infected animals, depending on the strain of amoebae, have revealed the presence of trophozoites of *Acanthamoeba* spp. between the cell junctions [26], inflammatory foci, fibrotic-like changes [25], petechiae and even necrotic changes in renal tubules and glomeruli [27]. To date, *Acanthamoeba* spp. have been found to increase the expression of Toll-like receptor 2 (TLR2) in the host kidney; TLRs are significant components of the innate immune response that are responsible for recognizing pathogen-associated molecular patterns (PAMPs) [28]. Additionally, increased expression and activity of matrix metalloproteinases -2 and -9 (MMP-2 and MMP-9) were observed in the kidneys of hosts with systemic acanthamoebiasis [10]. MMPs are considered to be some of the most important proteins degrading the components of the extracellular matrix (ECM), which affects physiological and pathological processes. It has been shown that MMPs can be activated at the level of gene expression and proenzyme activation through excessive production of ROS. Moreover, ROS together with MMPs can induce cell apoptosis by targeting apoptotic proteins such as Bax, Bcl-2 and Cas3 [29]. Research results obtained so far suggest that *Acanthamoeba* spp. may provoke the increased production of ROS and the modulation of the apoptosis process in the host kidneys. In this context, the study reported here aimed to confirm or exclude the roles of oxidative stress and increased apoptosis in the kidneys of hosts with systemic acanthamoebiasis.

*Acanthamoeba* sp. disturb the pro-antioxidative balance in the lungs as well as in the eyes of the host [30, 31]. In the present study, upregulation of NOX2 and NOX4 gene expression in immunocompetent mice infected with *Acanthamoeba* sp. was noted at 16 and 24 dpi. At the same time, increased ROS levels were determined to activate antioxidant defense mechanisms, including Nrf2 gene expression. In immunosuppressed mice infected with *Acanthamoeba* sp. (group AS), NOX2 gene expression was increased from the beginning of infection up until the last day of experiment, while activation of the Nrf2 gene was delayed and observed to occur up to 16 dpi. As reported in the scientific literature, dexamethasone, a corticosteroid, sensitizes cancer stem cells to chemotherapeutic agents by suppressing Nrf2 expression [32]. In this context, our results may indicate the importance of prompt diagnosis and rapid implementation of anti-*Acanthamoeba* treatment in immunosuppressed patients.

Imbalance in the redox state has also found in other parasitic diseases. Sharma et al. [33] reported an increased level of lipid peroxidation product malondialdehyde (MDA) in mouse kidneys infected with *Plasmodium* sp. Nanda et al. [34] found that the MDA level was significantly higher in the serum of patients infected with *Plasmodium* sp. and with acute renal failure compared to those with uncomplicated malaria. These authors also suggested that serum MDA levels in patients with malaria may be used as a marker of the severity of tissue damage. Baldissera et al. [35] reported that *Trypanosoma evansi* also causes lipid peroxidation in the renal tissue of experimentally infected rats, altering the antioxidant-oxidant status. Taking into account helminths, Oliveira et al. [36] observed a severe redox imbalance in the kidneys of mice infected with *Schistosoma mansoni*. It has also been reported that some pathogens developed a strategy that consists of disabling NOX assembly to subvert exposure to oxidants; for example, in *Leishmania* sp. infections, there is an impaired assembly of NOX2 [37–39] and activation of Nrf2 [40]. Reverte et al. [41] found that Nrf2 protects *Leishmania* sp. from ROS. It is also confirmed that Nrf2 activation in *Leishmania amazonensis* infection leads to parasite survival and disease progression [42]. It is possible that *Acanthamoeba* sp. act similarly to *Leishmania* sp. and that Nrf2 gene activation leads to parasite survival and disease progression [42, 43]. It is worth noting that in a study on the kidneys of mice in which Nrf2 gene expression was elevated, the morphological changes in the kidneys were the most visible in the histopathological examination [25].

Oxidative stress is one of the factors leading to increased apoptosis. Physiological apoptosis in the kidneys occurs but has only been observed to occur at a relatively low level. In cases of kidney damage, physiological apoptosis can be significantly intensified, leading to the development of a wide variety of diseases of this organ [21]. Cases in which modulation of apoptosis occurred in host cells during parasite infection have been described in the literature. Some parasites are able to inhibit apoptosis in host cells by accelerating the death of immunologically competent cells [44]. In protozoan parasitic diseases, increased apoptosis has been found in hosts infected with *Plasmodium* sp. and *Leishmania* sp. Wichapoon et al. [45] and Elias et al. [46] observed higher expression of Cas3 in the kidneys of patients infected with *Plasmodium falciparum* and in the kidneys of mice infected with *Plasmodium* sp., respectively. Kumar et al. [47] also noted a significantly higher level of Cas3 mRNA and Cas3 activity in the kidneys of *Leishmania donovani*-infected mice than in the control group of animals. On the other hand, Solano-Gálvez et al. [48] found that *Leishmania* sp. can display various strategies, including apoptosis inhibition, to downregulate host cell defense mechanisms in order to perpetuate infection. It has been shown that *L. donovani* causing visceral leishmaniasis can activate the expression of Bcl2, which in turn leads to the inhibition of nitric oxide (NO) production and enhances survival of the parasite.

*Acanthamoeba* spp. have been shown to induce apoptosis in extracerebral infections. A sequence of events occurs in the course of AK involving the production of MMPs that degrade membranes and induce cytolysis and apoptosis of corneal cells [49–51]. In our study, proapoptotic Bax protein concentration and/or gene expression were lower in the kidneys of immunocompetent mice infected with *Acanthamoeba* sp. than in the control group of mice. We also observed an increased level of the antiapoptotic Bcl-2 gene and/or Bcl-2 protein at 8 and 24 dpi. Cas9 and Cas3 gene expression increased in group A at 16 dpi, but the protein expression of caspases decreased at 24 dpi, with the decrease being statistically significant. In immunocompetent hosts, *Acanthamoeba* spp. do not lead to dysregulation of the Bax/Bcl-2 ratio, and in long-term infection, they even inhibit apoptosis of host renal cells.

The apoptosis mechanism in immunosuppressed *Acanthamoeba* sp. infected mice differed from that in immunocompetent mice. Proapoptotic Bax protein expression was higher in all of the immunosuppressed groups, while antiapoptotic Bcl-2 protein expression was lower in all of the immunosuppressed groups. This difference is likely to be due to the administration of MPS. It has been reported that MPS increased the gene expression of Bax and decreased the gene expression of Bcl-2 [52]. Pandey et al. [53] observed a reducing load of *L. donovani* when specific Bcl-2 inhibitors were used. These authors reported that the anti-apoptotic effect was reversed and that NO levels increased. In our study, we did not check the load of *Acanthamoeba* sp. in the host; as reported in one study, amoebae were re-isolated from more immunocompetent animals than immunosuppressed animals, but the difference was only one individual [25]. In immunosuppressed mice infected with *Acanthamoeba* sp., we also observed that Cas9 was activated at all time points. Cas3 was also noticeably activated at all time points. In the kidneys of immunosuppressed hosts, infection with *Acanthamoeba* sp. leads to increased apoptosis by the intrinsic pathway. However, in future studies, it will also be important to check the expressions of proteins involved in the extrinsic pathway of apoptosis.

**Fig. 6:**
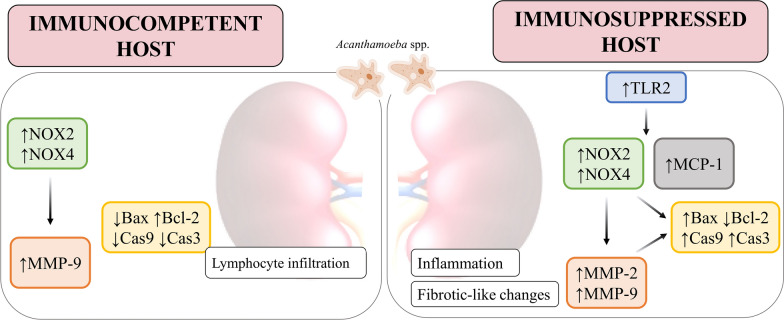
Mechanisms in the kidneys of mice infected with *Acanthamoeba* sp. In the immunocompetent mice, NOX2 and NOX4 gene expression were upregulated. Increased ROS levels activate antioxidant defense mechanisms and Nrf2 gene expression and/or protein concentration. NADPH oxidases probably activate MMP9 [10]. In the histopathological examination of the kidneys of immunocompetent infected mice, lymphocyte infiltration and mitotic figures were found [22]. In the immunosuppressed mice, higher expression of TLR2 was observed [28]. TLR2 probably activates NOX2 and NOX4 as well as MCP-1. Increased ROS levels increased Nrf2 gene expression; however, Nrf2 protein concentration did not change. NADHP oxidases probably activate MMP2 and MMP9 [10]. Interaction between NOX4 and TLR2 together with higher levels of MMPs leads to increased cell apoptosis in the kidneys. In the histopathological examination of the kidneys of immunosuppressed infected mice, poorly visible Bowman’s capsules and a lighter staining of the nuclei and cytoplasm of tubular cells were noted [22]. Cas3, Caspase 3; Cas9, caspase 9; MCP-1, monocyte chemoattractant protein 1; MMP9, matrix metalloproteinase 9; NOX2, NADPH oxidase 2; NOX4, NADPH oxidase 4; Nrf2, nuclear erythroid 2-related factor; ROS, reactive oxygen species; TLR2, Toll-like receptor 2

The study described here is the first to address the mechanisms occurring in the kidneys of hosts infected with *Acanthamoeba* sp. The kidneys are organs with a key detoxification process. Through filtering the blood, they can be attacked by parasites that are circulating in the blood. Most of the parasites cause changes in the kidneys, which are usually unnoticed because they are masked by extrarenal manifestations. The exact mechanisms of kidney injury caused in parasitic infections are poorly known in many cases, resulting in major difficulties to provide specific therapeutic interventions [10]. Therefore, research on the pathophysiology of kidney infection by parasites is very significant. Figure 6 provides a summary of the results of the present study on mechanisms in the kidneys infected with *Acanthamoeba* sp., showing increased gene expression and/or protein concentration of NOXs. The contact of *Acanthamoeba* sp. with the host kidney cell surface and/or the oxidative burst caused by elevated levels of NOXs boosted the antioxidant response enhanced by the Nrf2 pathway. In immunocompetent hosts, the antioxidant response was probably sufficient to sustain a redox state and therefore an increased apoptosis rate was not observed. Interestingly, in the group of hosts with normal immunity, *Acanthamoeba* sp. inhibited the apoptosis of kidney cells. In the immunosuppressed hosts, the antioxidant response was not sufficient to sustain a redox state, and even though the Nrf2 gene expression in the group of hosts with reduced immunity increased, the protein concentration of Nrf2 was lower. Increased apoptosis by the intrinsic pathway was noted in this group of animals. Based on results by Yang et al. [15], we suspect that increased apoptosis and ROS production in the kidneys of immunosuppressed mice infected with *Acanthamoeba* sp. are mediated by interaction between NOX4 and TLR2. When a higher level of apoptosis (based on higher activation of cleaved Cas3) was noted, only a higher protein concentration of NOX4 was observed. Taking into account TLRs, both TLR2 and TLR4 changes were examined in the kidneys of hosts infected with *Acanthamoeba* sp., but statistically significant differences were observed only in TLR2 expression [28].

This study has a number of limitations. First, the inoculum contained a variable number of amoeba trophozoites. Thus, the mice were not infected with the same number of parasites and, consequently, the host’s response to the amoebas may have differed. Secondly, monoxenic cultivation of amoebae was used and thus the inoculum contained inactivated bacteria. There was no group of control animals that received only deactivated bacteria in the inoculum. Thirdly, drug concentration in the blood of mice was not measured. Immunosuppression was assessed only by histopathological examination of the spleen and differences in the level of lymphocytes and cytokines between control groups (group C vs group CS) [54].

## Conclusions

The results of the present study showed that the mechanisms of oxidative stress and apoptosis in the host organisms vary depending on the immunological status of the host. In immunocompetent mice, the balance between NOXs and Nrf2 was probably preserved; therefore, the apoptosis rate was not higher. Interestingly, in the group of hosts with normal immunity, *Acanthamoeba* spp. inhibited apoptosis of the kidney cells. In the immunosuppressed mice, the antioxidant response through Nrf2 was probably not sufficient to sustain a redox state; in this group of animals, increased apoptosis by the intrinsic pathway was noted.

## Data Availability

The data supporting the findings of the study must be available within the article and/or its supplementary materials, or deposited in a publicly available database.

## Abbreviations

A: Immunocompetent *Acanthamoeba* sp.-infected mice
AK: *Acanthamoeba* keratitis
AM22: Amoebic strain no. 22
AS: Immunosuppressed *Acanthamoeba* sp.-infected mice
C: Immunocompetent uninfected control group mice
Cas3: Caspase 3
Cas9: Caspase 9
CS: Immunosuppressed uninfected control group mice
dpi: Days post-infection
ECM: Extracellular matrix
MDA: Malondialdehyde
MMPs: Matrix metalloproteinases
MPS: Methylprednisolone sodium succinate
mRNA: Messenger RNA
NOX2: NADPH oxidase 2
NOX4: NADPH oxidase 4
Nrf2: Nuclear erythroid 2-related factor
PAMPs: Pathogen-associated molecular patterns
ROS: Reactive oxygen species
qRT-PCR: Quantitative reverse transcription PCR
TLR2: Toll-like receptor 2
TLR4: Toll-like receptor 4
